# Efficiency and immunogenicity of lipid nanoparticle-mediated cardiac mRNA delivery are lipid composition-dependent

**DOI:** 10.1016/j.omtn.2026.102939

**Published:** 2026-04-22

**Authors:** Maria C.I. Labonia, Pol Escudé Martinez de Castilla, Petra H. van der Kraak, Maike A.D. Brans, Qiangbing Yang, Zhiyong Lei, Saskia C.A. de Jager, Willemijn S. de Voogt, Raymond M. Schiffelers, Joost P.G. Sluijter, Pieter Vader

**Affiliations:** 1 Department of Cardiology, Laboratory of Experimental Cardiology, University Medical Center Utrecht, Utrecht, the Netherlands; 2 CDL Research, University Medical Center Utrecht, Utrecht, the Netherlands; 3 UMC Utrecht Regenerative Medicine Center, Circulatory Health Research Center, University Medical Center Utrecht, Utrecht University, Utrecht, the Netherlands

**Keywords:** MT: Delivery Strategies, mRNA therapeutics, lipid nanoparticles, delivery, immunogenicity, cardiac

## Abstract

The efficiency and safety of modified messenger RNA (modRNA) delivery into the heart using lipid nanoparticles (LNPs) remain undetermined. We previously demonstrated that modRNA encapsulated in C12-200 LNPs outperforms current state-of-the-art intramyocardial modRNA delivery methods. Surprisingly, C12-200 LNPs triggered robust local immune cell activation 5 days post-injection, which was not evident on day 1. To investigate whether this immune response is driven by LNP composition or modRNA transfection efficiency, we systematically compared cardiac transfection efficiency, off-target biodistribution, and immunogenicity of modRNA formulated with clinically validated LNPs from Onpattro, BNT162b2/Comirnaty, and mRNA-1273/Spikevax. All tested formulations outperformed C12-200 in cardiac delivery, with mRNA-1273 and Onpattro showing markedly reduced off-target accumulation in the liver and spleen. Histopathological analysis revealed formulation-dependent immune cell infiltration, most pronounced with C12-200. C12-200 significantly elevated cytokine levels in both serum and heart tissue, whereas Onpattro increased cytokine levels mainly locally. In contrast, cytokine levels in animals treated with BNT162b2 and mRNA-1273 were comparable to those in PBS-treated controls. Importantly, cardiac transfection efficiency did not correlate with cytokine induction or histopathological changes, indicating that LNP-driven immunogenicity is independent of transfection efficacy. These findings provide a foundation for refining LNP formulations to optimize cardiac modRNA delivery while minimizing immune-related adverse effects.

## Graphical abstract

**Figure:**
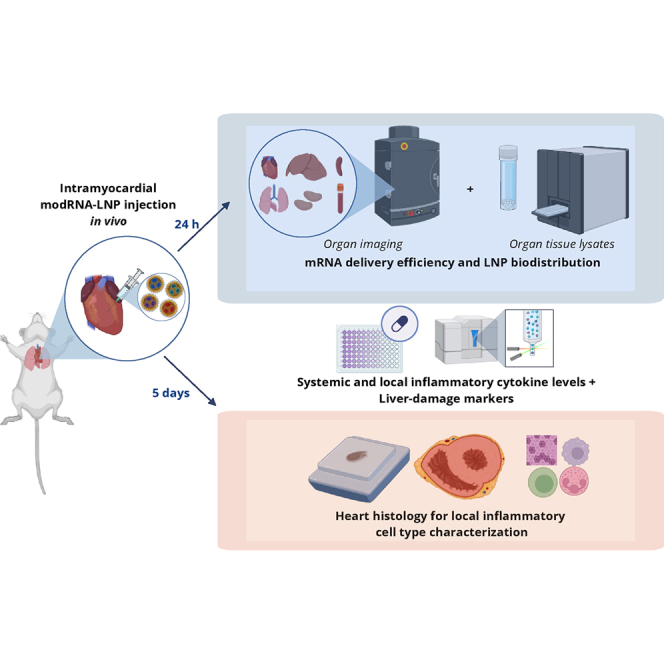

## Teaser

Vader and colleagues compare clinically used lipid nanoparticle formulations for cardiac delivery of modified mRNA. They show that delivery efficiency and immune activation are uncoupled, revealing formulation-dependent immunogenicity. These findings highlight strategies to improve the safety and targeting of cardiac mRNA therapeutics.

## Introduction

Heart failure is a chronic and progressive condition in which the heart is unable to pump blood efficiently enough to meet the body’s needs. It affects millions of people worldwide and is a leading cause of mortality and morbidity.^1^ Life expectancy of patients with end-stage heart failure is drastically reduced, and the only therapeutic options for these patients are a mechanical support device or a heart transplant. Modified messenger RNA (modRNA) therapeutics have the potential to directly address the loss of functional heart tissue by encoding proteins that can promote the recovery of cardiomyocytes, reduce inflammation, inhibit fibrosis, or stimulate angiogenesis.^2^^,^^3^ Importantly, for modRNA applications it is essential to use appropriate delivery systems to protect and ensure effective modRNA delivery into target cells, as modRNA is inherently unstable and prone to degradation by RNases.

During the severe acute respiratory syndrome coronavirus 2 (SARS-CoV-2) pandemic, modRNA vaccines were rapidly developed, successfully implemented to treat millions of people worldwide, and proven to be both effective and safe.^4^^,^^5^^,^^6^ This success highlighted the tremendous therapeutic potential of modRNA delivery and spurred research into various non-vaccine therapeutic directions. These mRNA coronavirus disease 2019 (COVID-19) vaccines made use of lipid nanoparticles (LNPs) as a delivery system to protect the fragile mRNA molecule and facilitaxte cell entry. LNPs are primarily composed of different types of lipids, including cationic or ionizable lipids, phospholipids, cholesterol, and PEGylated lipids.^7^ Noteworthy, it has been reported that cationic/ionizable lipids, which are a key LNP component for functional RNA encapsulation and delivery, exhibit a pro-inflammatory role.^8^ For different routes of LNP administration (intradermal, intranasal, and intramuscular), a robust immune activation response, characterized by neutrophil infiltration and triggering of multiple inflammatory cytokine pathways, has been described.^9^ Moreover, LNP-induced systemic inflammation exacerbates the inflammatory response in a mouse model of acute inflammation.^10^ Hence, while the locally induced immune activation of LNPs is a desirable characteristic for vaccine applications due to its adjuvant activity, it may hinder therapeutic delivery of LNPs for other indications. To address this challenge, current research aims to optimize LNP formulations by using more potent or less immunogenic lipids.

We have previously shown the potential of modRNA LNP delivery for cardiac applications by comparing it to the current state-of-the-art method, namely intracardiac administration of modRNA in sucrose-citrate buffer solution.^11^ Notably, we observed no histological alterations associated with immune response in the heart 24 h after injection. In the current study, however, we evaluated the local immune response elicited by the cardiac delivery of C12-200 modRNA LNPs 5 days after administration, revealing robust immune cell infiltration at the injection site. To identify less immunogenic modRNA LNP formulations, we evaluated the transfection efficiency and immunogenicity of various LNP formulations following cardiac administration. We tested the Onpattro formulation, used clinically to treat hereditary transthyretin amyloidosis (hATTR), as well as the lipid formulations used in the BNT162B2/Comirnaty and mRNA-1273/Spikevax COVID-19 vaccines, with the main distinction being the type of ionizable lipid used: D-Lin-MC3-DMA, ALC-0315, and SM-102, respectively. This study aimed to determine whether modRNA LNP cardiac transfection efficiency and immunogenicity upon local administration are dependent on lipid composition.

## Results

### C12-200 modRNA LNPs induce a local cardiac immune response upon intramyocardial administration

**Figure 1:**
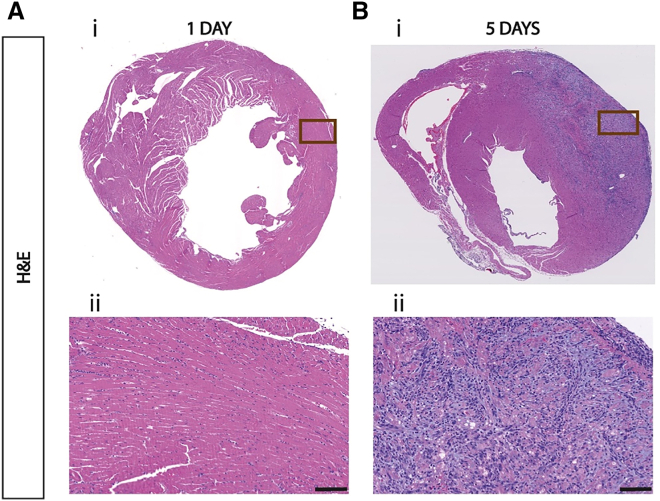
A robust local immune response is apparent 5 days after local intramyocardial administration of modRNA-LNPs H&E staining of transversal heart sections at 1 (A) and 5 days (B) after intramyocardial administration of C12-200 LNPs (C12-200 40%/Chol. 43.5%/DOPE 15%/DMG-PEG 1.5%) containing 4 μg of LNP-encapsulated modRNA. The histological sections of the hearts show marked differences in the local immune response to mRNA-LNP administration. Representative areas of the treated myocardium are presented by squares in a*i* and b*i* and magnified in a*ii* and b*ii*, respectively. Scale bars, 100 μm.

Previously, we reported that modRNA LNPs outperformed modRNA in citrate buffer for cardiac RNA delivery after intramyocardial administration.^11^ At 24 h after local administration, no visible immune cell infiltration was observed in histological analysis. However, by extending our follow-up to 5 days post-injection of C12-200 LNP-mediated cardiac modRNA delivery, histopathological analysis revealed robust immune cell infiltration at the injection site in the left ventricular wall, surprisingly contrasting with our findings at 1 day post administration (Figures 1A and 1B). This indicates that local administration of modRNA C12-200 LNPs induces a local immune response that only becomes histologically apparent at a later time point than 24 h after administration.

### LNP size, polydispersity, surface charge, and modRNA encapsulation efficiency are similar among the tested LNP formulations

**Figure 2:**
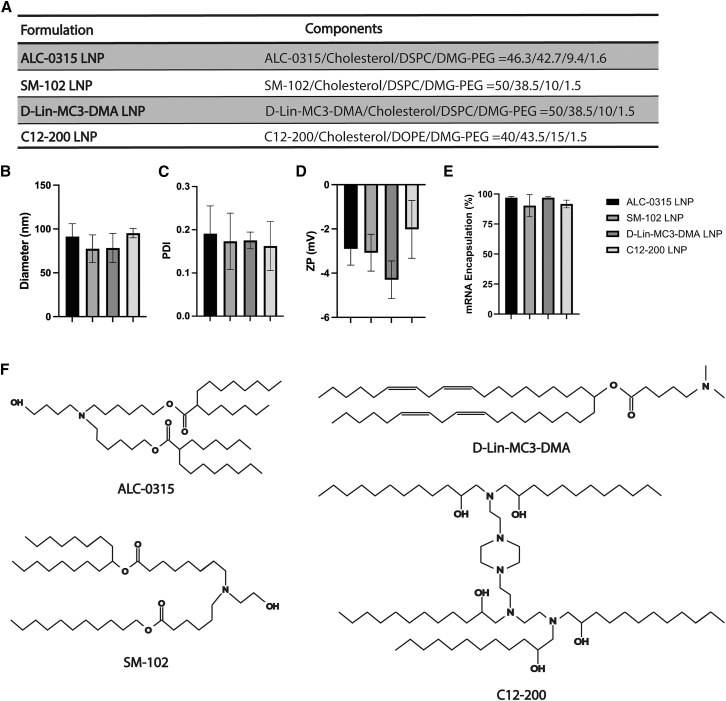
LNP size, polydispersity, surface charge, and modRNA encapsulation efficiency are similar among the tested LNP formulations (A) Overview of the lipid molar compositions of the four tested LNP formulations. LNP characterization consisted of LNP particle size (B), polydispersity index (PDI) (C), particle surface ζ-potential (ZP) (D), and modRNA encapsulation efficiency (E). Data represent the mean ± SD of three measurements. (F) Chemical structures of ionizable lipids used in this study.

To explore whether the observed immunogenic response was specifically associated with C12-200 LNPs, we proceeded to evaluate other LNP formulations corresponding to Onpattro (D-Lin-MC3-DMA ionizable lipid),^12^^,^^13^ BNT162B2 (ALC-0315 ionizable lipid),^6^ and mRNA-1273 (SM-102 ionizable lipid),^14^^,^^15^ for intramyocardial modRNA administration. After formulating the LNPs, nanoparticles were characterized by evaluating nanoparticle size, polydispersity index (PDI), surface charge, and modRNA encapsulation efficiency. Lipid molar compositions of the LNP formulations tested are indicated in Figure 2A. For all generated LNPs, sizes ranged between 60 and 100 nm in diameter (Figure 2B), and PDI ranged between 0.1 and 0.25, indicating a homogeneous nanoparticle size distribution (Figure 2C). The surface charge, measured as zeta potential, was maintained consistently in the range between −5 and −1 mV among LNP formulations (Figure 2D). Furthermore, our LNPs exhibited encapsulation efficiencies ranging between 90% and 98% (Figure 2E). These characterizations align with published data on these modRNA LNP formulations,^15^^,^^16^^,^^17^ suggesting that typical LNP characteristics are maintained across these LNP formulations. Chemical structures of ionizable lipids used in this study are presented in (Figure 2F).

### ALC-0315, SM102, and D-lin-MC3-DMA LNP formulations display enhanced cardiac modRNA delivery compared to C12-200 LNPs

Subsequently, we compared cardiac modRNA delivery efficiency, off-target delivery, and both local and systemic inflammatory responses of the characterized LNP formulations following intramyocardial administration in healthy mice.

**Figure 3:**
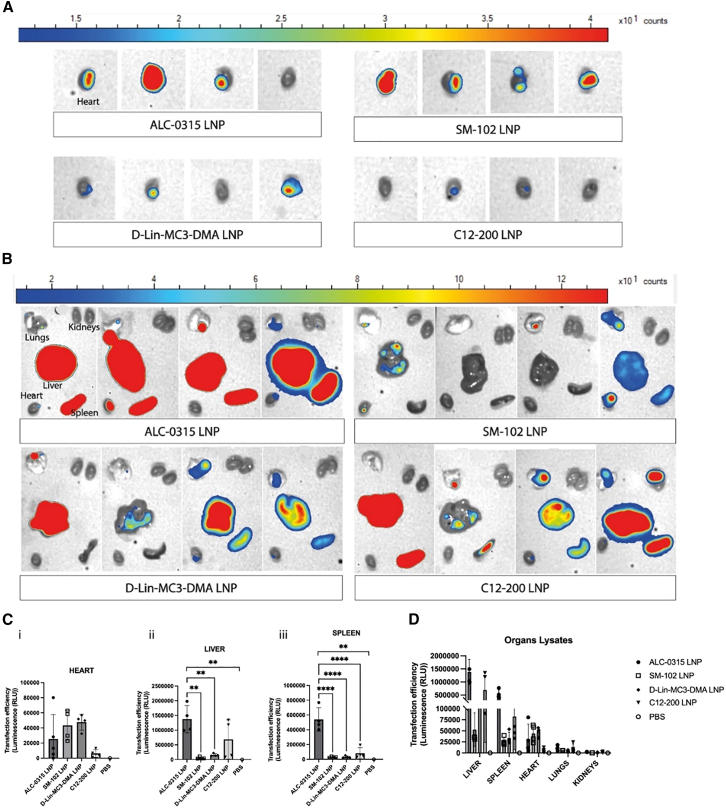
The tested LNP formulations are effective for cardiac modRNA delivery, with SM-102 LNPs presenting the most favorable profile Cardiac delivery efficiency of luciferase-encoding modRNA was measured by luminescence imaging of hearts (A) and other organs (B) 24 h after intramyocardial administration of modRNA LNPs. Luminescence quantification was also performed in organ lysates (C and D). Data represent the mean ± SD of *n* = 4 for the treatment groups and *n* = 1 for the PBS group. ∗∗∗∗, *p*-value <0.0001; ∗∗∗, *p*-value <0.001; ∗∗, *p*-value <0.01; ∗, *p*-value <0.05; blank: no significant difference.

Twenty-four hours after local administration of luciferase-encoding modRNA LNPs, we performed luminescence imaging on isolated hearts and other organs (heart, spleen, lungs, liver, and kidneys). By comparing luminescence levels, it was evident that hearts treated with ALC-0315, SM-102, and D-Lin-MC3-DMA LNPs presented the highest luciferase modRNA translation levels compared to hearts treated with C12-200 LNPs (Figure 3A). Interestingly, organ luminescence imaging indicated the highest modRNA translation levels in the livers and spleens of mice treated with ALC-0315 and C12-200 LNPs, while SM-102 LNPs showed significantly lower off-target organ delivery (Figures 3B; Figure S1).

To quantify luciferase activity at the tissue level, organs were processed into tissue homogenates (Figures 3C and 3D). Quantitative results showed that all the newly tested LNPs were considerably more efficient than C12-200 LNPs in delivering modRNA to the heart (Figure 3C*i*). Furthermore, quantitative analysis of liver and spleen homogenates confirmed that ALC-0315 LNPs presented the highest off-target delivery among the tested LNP formulations, while SM-102 LNPs presented the lowest off-target delivery (Figure 3C*ii,iii*). Among the analyzed organs, luciferase expression showed the lowest levels in the kidneys, followed by the lungs, with expression levels comparable across LNP formulations (Figure 3D). From these outcomes, we concluded that SM-102 LNPs display the most promising characteristics for modRNA delivery to the heart after local administration.

### Immune activation triggered by modRNA LNP cardiac local administration is dependent on LNP lipid composition

**Figure 4:**
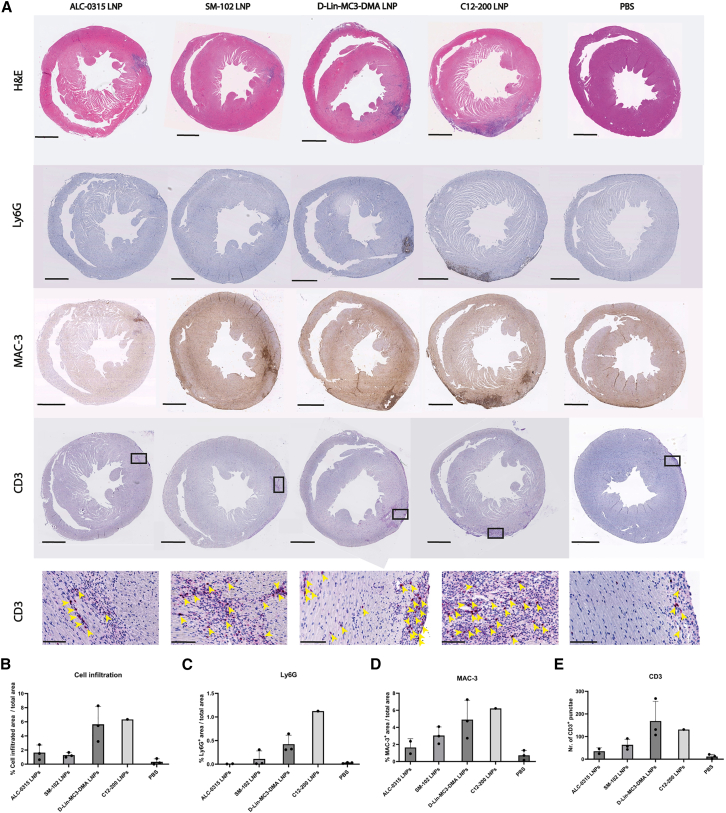
LNPs induce different degrees of cardiac inflammatory responses, characterized by neutrophil, macrophage, and T cell infiltration at the injection site 5 days after modRNA-LNP intramyocardial administration (A) Representative images of H&E, Ly6G, MAC-3, and CD3 stainings of mouse heart sections collected 5 days after local injection of 4 μg modRNA encapsulated in LNPs or PBS into the left ventricular wall. (B) Quantification of maximum cell infiltration in H&E-stained heart sections. (C) Quantification of the area of Ly6G^+^ infiltrating cells in heart sections, corresponding to neutrophils. (D) Quantification of the area of MAC-3^+^ infiltrating cells in heart sections, corresponding to macrophages. (E) Quantification of the number of CD3^+^ infiltrating cells in heart sections, corresponding to T cells. At least three histological sections per staining and per animal were analyzed. For each staining, the maximum infiltrated area (B, C, D) or maximum number of positive cells (E) per animal was plotted. All scale bars in (A) represent 1 mm, except for the zoomed-in CD3 images in the bottom row, which represent 100 μm. Data represent the mean ± SD of *n* = 1–3 animals (B, C, D, E).

After evaluating modRNA LNP cardiac delivery efficacy, we proceeded with histopathological analysis of hearts at 5 days post administration to study potential local immune responses, as described above for C12-200 LNPs (Figure 1). While the mechanism by which LNPs activate immune cells remains unclear, multiple studies show they trigger the innate immune system, with cytokine secretion amplifying communication between immune cells.^8^^,^^18^^,^^19^^,^^20^^,^^21^ Recent studies have shown that empty LNPs already affect the phagocytic function of peripheral blood mononuclear cells (PBMCs) and stimulate phagocytosis.^19^ General cell infiltration was first assessed by hematoxylin and eosin (H&E) staining, while specific immune cell populations were identified using the neutrophil marker Ly6G, the macrophage marker MAC-3 (also known as CD107b or LAMP2),^22^ and the T cell marker CD3 (Figure 4A). Scans of heart sections treated with different modRNA LNPs reflected varying degrees of immune cell infiltration, all occurring surrounding the injection site.

Quantification of cell infiltration using H&E staining at 5 days post-injection demonstrated marked cellular infiltration in hearts treated with C12-200 and D-Lin-MC3-DMA LNPs (Figures 4A and 4B*)*. In contrast, ALC-0315 and SM-102 LNP-treated hearts exhibited moderate infiltration, while phosphate-buffered saline (PBS)-injected controls showed minimal infiltration, primarily localized around the injection sites. Immunohistochemical analysis of Ly6G staining revealed a high abundance of neutrophils in the C12-200 group, a moderate abundance in the D-Lin-MC3-DMA group, low abundance in the SM-102 group, and negligible neutrophil presence in the ALC-0315 and PBS groups (Figures 4A–4C). Immunohistochemical staining for MAC-3 revealed predominant macrophage infiltration at the injection site in all LNP-treated groups, with the highest levels observed for C12-200 LNPs, followed by D-Lin-MC3-DMA LNPs, and moderate infiltration for SM-102 and ALC-0315 LNPs (Figures 4A–4D). Hearts treated with PBS also presented some degree of macrophage infiltration, highlighting the intrinsic immune-activating injury caused by invasive local administration. CD3 staining revealed limited but detectable T cell infiltration across all LNP-treated groups (Figures 4A–4E), most prominently for D-Lin-MC3-DMA and C12-200 LNPs, and to a lower degree for ALC-0315 and SM-102 LNPs, and negligible infiltration in PBS controls. T cells were primarily localized to the same regions surrounding the injection site in the left ventricular wall. Thus, from this histopathological analysis, we conclude that all modRNA LNPs induce some degree of local immune activation after administration, but the extent varies largely depending on LNP composition.

### modRNA-LNP lipid composition influences inflammatory cytokine response and liver transaminases levels following cardiac administration

**Figure 5:**
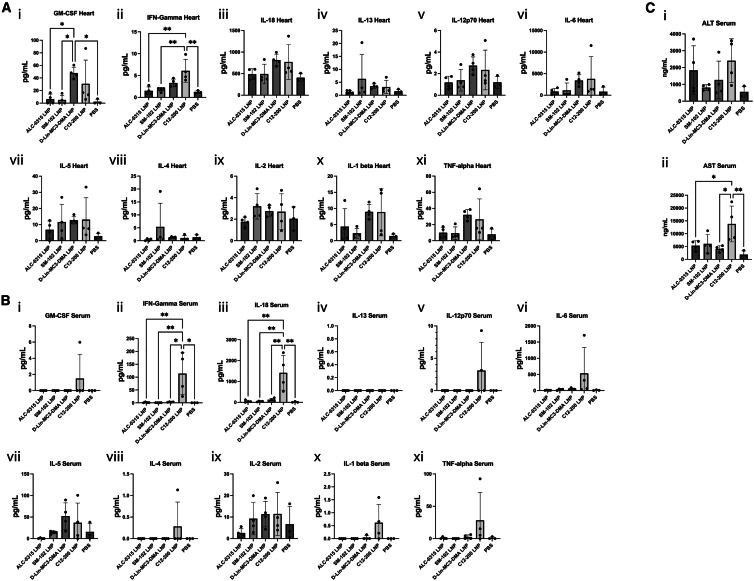
ALC-0315 LNPs and SM-102 LNPs do not induce upregulation of pro-inflammatory cytokines in hearts or serum 24 h post local administration Quantification of pro-inflammatory cytokines in hearts (A) and in serum (B) 24 h after local LNP administration in the heart. (C) Quantification of liver transaminases ALT (C*i*) and AST (C*ii*) in serum. Abbreviations are as follows: GM-CSF, granulocyte macrophage colony stimulating factor; IFN, interferon; IL, interleukin; TNF, tumor necrosis factor; ALT, alanine transaminase; AST, aspartate aminotransferase. Data represent the mean ± SD of *n* = 4 for treatment groups and *n* = 3 for PBS groups. ∗∗∗∗, *p*-value <0.0001; ∗∗∗, *p*-value <0.001; ∗∗, *p*-value <0.01; ∗, *p*-value <0.05; blank: no significant difference.

To further characterize the observed inflammatory response, we examined the presence and levels of various inflammatory cytokines in the treated hearts and serum 24 h after local cardiac administration of modRNA LNPs. This time point was chosen as cytokine levels in tissues may decay at 1 day post modRNA LNP local administration, as Li et al. reported for intramuscular modRNA LNP administration.^23^ Quantitative analysis showed no statistically significant differences in any of the cytokine levels in hearts treated with ALC-0315 LNPs or SM-102 LNPs, compared with the PBS control (Figure 5A). In contrast, hearts treated with D-Lin-MC3-DMA or C12-200 LNPs showed a clear increasing trend in GM-CSF, IL-6, IL-1β, and TNF-α levels, although the differences compared with the PBS control group were not always statistically significant. Furthermore, hearts treated with C12-200 LNPs presented a significant upregulation of IFN-γ compared with SM-102 LNPs, ALC-0315 LNPs, and the PBS control group. These datasets confirm our previous observations that ALC-0315 LNPs and SM-102 LNPs are less immunogenic than D-Lin-MC3-DMA LNPs and C12-200 LNPs.

In serum, none of the 11 analyzed cytokines were upregulated by treatment with D-Lin-MC3-DMA, ALC-0315, or SM-102 LNPs. However, significantly upregulated levels were detected for IFN-γ and IL-18 in the serum of mice treated with C12-200 LNPs, and a clear trend of increased IL-6, IL-1β, TNF-α, and IL-12 p70 levels could be observed (Figure 5B). Thus, across the different LNP treatments, both local and systemic levels of pro-inflammatory cytokines at day 1 correlate with levels of local immune cell infiltration at day 5. Mechanistically, it seems that a variable innate immune response is triggered by the different LNPs, thereby inducing secretion of pro-inflammatory cytokines that promote infiltration of activated leukocytes into the damaged myocardium.

Finally, taking into account that the liver was identified as the main off-target organ for LNPs, we also measured liver transaminases alanine transaminase (ALT) and aspartate aminotransferase (AST) as surrogate markers for liver toxicity in serum at 24 h after injection. We found a significant upregulation of AST in mice treated with C12-200 LNPs compared with mice treated with D-Lin-MC3-DMA LNPs, ALC-0315 LNPs, or PBS, suggesting that C12-200 LNPs, but not the other LNP formulations, induced some degree of liver toxicity (Figure 5C). Altogether, these outcomes indicate that LNP composition determines LNP immunogenicity and toxicity after local cardiac administration.

## Discussion

Despite the considerable public health burden of cardiac diseases and the high potential of nucleotide-based therapies, there is still a lack of available therapies to treat the diseased heart. modRNA therapeutics have the potential to directly address the underlying pathological problem by encoding proteins that can target the affected mechanisms that lead to heart disease. The most prominent examples of mRNA-based clinical applications are the mRNA vaccines for COVID-19 (from Pfizer-BioNTech and Moderna), which have proven to be effective and safe in millions of individuals.^14^ Currently, it remains to be determined whether this LNP-mediated modRNA delivery strategy can be used to enable therapeutic modRNA delivery to the affected myocardium.

Aiming for LNPs targeting tissues other than the liver and spleen presents a major challenge, as off-target delivery to these organs is usually overwhelmingly high upon systemic administration.^24^ In this context, many research groups working on cardiac nucleic acid delivery choose the intramyocardial administration route, aiming for desirable local cardiac retention.^3^^,^^25^ However, this procedure is quite invasive, and our results suggest that it induces a certain basic level of local inflammation due to tissue disruption caused by needle puncture, further enhanced by LNP composition. Nevertheless, intramyocardial cell delivery has been shown to be clinically safe in patients undergoing elective coronary artery bypass surgery.^26^ Furthermore, from an efficiency perspective, optimized strategies for modRNA delivery to the heart are highly demanded, and LNPs (as modRNA delivery vectors) are presented as potential candidates.^11^ Importantly, when it comes to LNP administration to the heart, it is crucial to consider LNP cardiac cell delivery efficacy, tolerability, and off-target organ delivery as important variables.

We started our studies using C12-200 LNPs, which displayed efficient delivery of modRNA and no visible immune cell infiltration at the injection site at day 1 post-injection.^11^ However, in the current work, we discovered a proinflammatory infiltrate in the heart at a later time point (Figure 1). To subsequently identify less immunogenic LNP formulations, we tested three lipid formulations that had already been approved for other clinical applications, containing D-Lin-MC3-DMA, ALC-0315, or SM-102 ionizable lipids. The outcomes presented in this study on modRNA LNP delivery efficiency (Figure 3) and immunogenicity (Figures 4 and 5) upon local intramyocardial administration suggest that all three tested LNP formulations deliver modRNAs to the heart more efficiently than C12-200 LNPs, while inducing lower systemic inflammation and varying degrees of local immune activation and off-target organ delivery. Importantly, despite differences in molar lipid composition and N:P ratio, all LNPs displayed comparable size, PDI, surface charge, and encapsulation efficiency (Figure 2), suggesting that the observed biological differences are unlikely to be explained solely by gross physicochemical disparities. The differences between LNP formulations seem to mainly rely on their ionizable lipid component, although other formulation parameters, including molar lipid composition and N:P ratio, may also contribute. DOPE in the C12-200 LNP likely promotes membrane fusion and endosomal escape due to its cone-shaped geometry, which favors fusogenic behavior and DOPE has been suggested to enhance cytosolic mRNA release.^27^^,^^28^ However, its strong fusogenic behavior may also destabilize cellular membranes and contribute to increased innate immune activation, potentially amplifying the inflammatory response observed with C12-200 LNPs.

It has been reported that the ionizable lipid component is the main trigger of LNP immunogenicity, and current optimization efforts aim to increase its biodegradability.^29^^,^^30^ One of the structural components of ionizable lipids that determines LNP biodegradability is the ionizable lipid linker, which connects the head and tail groups of the lipid. Currently, most efforts to make ionizable lipids biodegradable involve incorporating ester bonds as linkers. In this study, C12-200 does not contain ester linkages, and although D-Lin-MC3-DMA contains an ester linkage, it may not be biodegradable, as the presence of a cleavable linker alone is not sufficient without evidence of its actual cleavage.^31^ In contrast, ALC-0315 and SM-102 ionizable lipids contain ester bonds that can be hydrolyzed by intracellular and tissue esterases,^32^ which may account for the lower levels of cell infiltration (evident from H&E staining), as well as reduced neutrophil, macrophage, and T cell infiltration, and a lower cytokine response compared with C12-200 and D-Lin-MC3-DMA LNPs. Based on our results, we hypothesize that the longer the ionizable lipid remains undegraded in tissue, the stronger the local immune response. A potential explanation for this phenomenon could be that undegraded lipids may be sensed by Toll-like receptors (TLRs) on macrophages and dendritic cells, thereby triggering inflammasome activation and the release of pro-inflammatory cytokines as part of the innate immune response.^18^ Future studies should directly assess ionizable lipid persistence and degradation in cardiac tissue (e.g., by liquid chromatography-mass spectrometry) to validate this proposed mechanism. Beyond biodegradability and lipid clearance, further optimization of ionizable lipid and LNP design may enhance the efficacy and tolerability of mRNA therapeutics, particularly for non-vaccine applications. Next-generation ionizable lipids with reduced inflammatory potential, optimized pKa values for efficient endosomal escape, and rapid clearance are being developed. Additional strategies include refining overall lipid composition, incorporating targeting ligands to enable selective organ or cell delivery after systemic administration, and exploring less invasive routes such as intracoronary infusion^33^ to improve tissue specificity and reduce systemic exposure.

Interestingly, C12-200 and D-Lin-MC3-DMA LNPs induced a robust local cytokine response, characterized particularly by secretion of GM-CSF, IFN-γ, and IL-18 within 24 h post-injection. These cytokines play central roles in innate immune cell recruitment and activation, with GM-CSF in particular acting as a potent stimulator of neutrophil expansion and migration.^34^ The upregulation of GM-CSF in C12-200 and D-Lin-MC3-treated groups 24 h post-injection aligns with the increased Ly6G-positive neutrophil infiltration observed histologically at day 5 (Figures 4A–4C), suggesting that neutrophil recruitment was initiated early and maintained over several days. Conversely, SM-102 and ALC-0315-treated hearts showed no significant GM-CSF elevation at 24 h and no considerable neutrophil infiltration at 5 days. To rule out the possibility that these less immunogenic LNPs elicited an earlier granulocytic response that we could have missed at day 5, we performed H&E staining on transversal heart sections 24 h after intramyocardial administration of SM-102 LNPs. The analysis revealed no significant cellular infiltration at this early time point, indicating that SM-102 LNPs do not trigger a major neutrophil response in the heart at any stage (Figure S2).

Our immunohistochemistry analyses showed abundant MAC-3^+^ macrophage recruitment (Figures 4A–4D) and limited T cell infiltration (Figures 4A–4E) at the injection site in all LNP-treated groups, with the strongest responses for C12-200 LNPs, followed by D-Lin-MC3-DMA LNPs, and moderate levels for SM-102 and ALC-0315 LNPs. PBS-injected hearts also exhibited some macrophage infiltration, likely reflecting innate immune activation caused by mechanical injury from intracardiac needle insertion. Previous evidence suggests that following tissue injury, neutrophils are the initial responders, whereas monocytes infiltrate later and differentiate into macrophages,^35^ likely explaining their predominance in the PBS group at 5 days post-injection. Moreover, macrophages are key mediators of nanoparticle uptake and processing, which supports their presence at the LNP injection site in the myocardium at this time point.^36^

To identify which cardiac cell types take up and translate mRNA after local LNP administration, we performed immunofluorescence staining. To capture immune cell infiltration, tissues were analyzed 5 days post-injection. At this time point, GFP expression was detectable in both cardiomyocytes and interstitial cells, with a highly localized distribution confined to the injection site in the left ventricular wall. This pattern was comparable across ALC-0315, SM-102, and D-Lin-MC3 formulations (S3a-bi). Thus, although overall transfection efficiency and immune activation differed among LNPs, our data do not indicate formulation-dependent differences in the cellular tropism of transfection. The detection of GFP-positive cardiomyocytes within the treated region supports the feasibility of this local mRNA delivery approach to drive expression of other proteins in the myocardium for therapeutic purposes.

We could also determine that the Ly6G-positive infiltrating cells, consistent with neutrophils, were GFP-negative (S3bii). This may reflect the timing of neutrophil recruitment relative to peak mRNA-LNP uptake and/or translation.

Although the exact mechanism by which immune cells sense LNPs is not fully comprehended yet, it is known that they activate various components of the innate immune system.^18^ Our results showed cytokine upregulation in heart homogenates or serum at 24 h post-administration in animals treated with C12-200 or D-Lin-MC3-DMA LNPs (Figure 5). In contrast, none of the 11 screened cytokines were upregulated in animals treated with SM-102 or ALC-0315 LNPs, compared with the PBS control group. In line with our cytokine analysis in heart lysates 24 h after administration of C12-200 and D-Lin-MC3-DMA LNPs (Figure 5A), recent studies have shown how *in vivo* administration of empty LNPs or LNPs encapsulating non-coding mRNAs can trigger production of the following cytokines: IL-1β,^9^ IL-6,^9^^,^^19^^,^^20^ GM-CSF,^9^ and TNF-α.^21^ Moreover, in a study where empty LNPs were intramuscularly administered to mice, an upregulation of IFN-γ was observed.^21^ Similarly, in our study, C12-200 LNPs, which were the most immunogenic LNPs tested, induced significant upregulation of IFN-γ both in the heart and in serum (Figure 5A*ii*,B*ii*). Notably, although C12-200 exhibited a lower ionizable lipid-to-RNA mass ratio compared with the other formulations, its multivalent structure results in a higher effective charge density per molecule, which may contribute to its distinct transfection efficiency and immunogenicity profile.

A limitation of the present study is that multiple formulation parameters differ between the evaluated LNP systems, including molar lipid composition, phospholipid species, and N:P ratio. Consequently, the relative contribution of each individual parameter to the observed differences in transfection efficiency and immunogenicity cannot be fully disentangled. In addition, although all formulations carried the same type and dose of modRNA, differences in transfection efficiency or cellular targeting may influence innate immune responses. Therefore, the respective contributions of lipid components and expressed mRNA cannot be definitively separated in the current experimental design. The study was conceived as a translational comparison of established LNP systems rather than a mechanistic dissection of individual formulation variables. Future studies systematically varying single parameters within a controlled formulation framework would help clarify their specific roles.

Although the ionizable lipids SM-102 and ALC-0315 already contain ester bonds that can be hydrolyzed by intracellular and extracellular esterases,^32^ there is still considerable scope for LNP optimization to achieve higher tolerability. Developing more potent and/or less immunogenic lipids, as well as optimizing LNP lipid composition, is crucial for balancing efficient cardiac delivery with minimal adverse immune reactions, offering a promising therapeutic alternative for the treatment of the diseased heart.

### Conclusion

In this study, we show that LNP lipid composition strongly influences modRNA delivery efficiency and immune activation in the heart. After local intramyocardial administration, SM-102 LNPs achieved robust cardiac modRNA expression with minimal off-target organ detections and did not induce major immune cell recruitment, unlike the more immunogenic C12-200 and D-Lin-MC3-DMA formulations. These outcomes strongly suggest that LNP lipid composition can determine cardiac delivery efficiency and off-target organ delivery after local intramyocardial administration. Variations in LNP components (ionizable lipids, cholesterol, phospholipids, and PEG-lipids) can significantly impact how LNPs interact with cardiac tissue and resident immune cells, potentially triggering inflammatory responses or altering the efficacy of the therapeutic payload. A deeper understanding of how different lipid components influence immunogenicity can guide the design of safer and more effective LNP-based therapeutics for cardiac applications.

## Materials and methods

### IVT mRNA synthesis

Firefly luciferase and EGFP mRNAs were generated in-house.^37^ The DNA sequences encoding luciferase and EGFP were cloned into a proprietary plasmid for mRNA production, featuring a T7 RNA polymerase promoter, a 5′ UTR derived from HIV gp160, a 3′ UTR from mouse α-globin, and a 120-nucleotide poly(A) tail. Capped mRNAs were synthesized using the VENI All-in-One mRNA Synthesis Kit (Leish Bio) following the manufacturer’s instructions. The reaction setup included 1× T7 reaction buffer, 10 mM DTT, 5 mM each of ATP, CTP, guanosine triphosphate (GTP), and N1-methylpseudo-UTP (m1ΨTP), 50 ng/μL of template DNA, and 5 mM of a cap 1 analog. Reactions were conducted at 37°C for 2 h, after which TURBO DNase (Thermo Fisher Scientific) was added at 2 U/μL and incubated for 15 min at 37°C to degrade residual DNA templates. Purification of the mRNAs was achieved through precipitation with an equal volume of precipitation solution, followed by two washes with cold 70% ethanol. The resulting mRNA pellets were dissolved in nuclease-free water. The concentrations of synthesized mRNAs were quantified using a DS-11 Spectrophotometer (DeNovix). Prior to analysis, 500 ng of each mRNA was heat-denatured and subjected to electrophoresis on a 1.5% agarose gel containing 0.01% (v/v) GelRed nucleic acid stain. A GeneRuler 1 kb Plus DNA Ladder (Thermo Fisher Scientific) was utilized as the molecular weight marker.

### LNP formulation

LNPs were produced through microfluidic mixing using a NanoAssemblr Benchtop system (Precision Nanosystems).^37^ The production involved combining a lipid solution in ethanol with an acidic aqueous phase containing the mRNA in 100 mM sodium acetate (pH 4.0). The formulation of the LNPs was performed at a flow rate ratio of 3:1 (aqueous to organic), with a total flow rate of 9.0 mL/min. Lipids were dissolved in 100% ethanol at concentrations between 5 and 20 mM. An N:P ratio of 6 was used to formulate SM-102 LNPs and ALC-0315 LNPs, whereas an N:P ratio of 10 was used for D-Lin-MC3-DMA LNPs (corresponding to ionizable lipid:RNA (w/w) ratios of 13, 14, and 19, respectively). For C12-200 LNPs, an N:P ratio of 15 (ionizable lipid:RNA (w/w) ratio: 10) was applied, calculated based on the assumption that five tertiary amine groups per lipid molecule are protonated at formulation pH. The selected lipid compositions and N:P ratios were based on previously reported or internally optimized formulations for each ionizable lipid, reflecting clinically established or validated conditions rather than enforcing identical formulation parameters across lipids. The LNP lipid molar compositions are indicated in Figure 2A. Immediately after production, the LNPs were dialyzed at 4°C in Slide-a-Lyzer dialysis cassettes G2 (20 kDa membrane cutoff) for 16–28 h against an excess of PBS (pH 7.4).

### LNP characterization

#### Dynamic Light Scattering for size and PDI measurement

The hydrodynamic diameter of the LNPs was measured using dynamic light scattering (DLS) with a Zetasizer Nano S instrument (Malvern Panalytical). A 4 mW HeNe laser operating at 633 nm was employed for these measurements. The LNPs were diluted in Dulbecco’s PBS (DPBS, pH 7.4), and scattering measurements were performed at an angle of 173° and a temperature of 25°C. Each measurement lasted 10 s and was repeated ten times, with each sample measured three times independently.

#### Zeta potential measurement

Zeta potential was measured using a Zetasizer Nano Z (Malvern Panalytical) after calibration with a Zeta Potential Transfer Standard (Malvern Panalytical). LNPs were diluted in 10 mM HEPES (pH 7.4), and each sample was measured at least three times independently.

#### mRNA quantification and encapsulation efficiency assessment

The total mRNA concentration was quantified using the Quant-It Ribogreen RNA Assay kit (Thermo Fisher Scientific) in the presence of 2% (v/v) Triton X-100 in TE buffer. Free mRNA concentration was measured separately in TE buffer alone. Lysed LNPs produced a fluorescent signal corresponding to the total mRNA (μg/μL), while non-lysed samples indicated the concentration of free, non-encapsulated mRNA (μg/μL). Calibration curves were prepared for both 2% Triton X-100 and TE buffer to accurately quantify mRNA concentration. The amount of encapsulated mRNA was determined by subtracting the free mRNA concentration from the total mRNA concentration. The percentage of encapsulated mRNA was then calculated using the formula: ((Total mRNA – Free mRNA)/Total mRNA) × 100.

### Animal experiments and tissue analysis

#### Ethical statement on animal experiments

All animal experiments were performed in compliance with ethical guidelines established by the Utrecht Animal Welfare Body and were approved under the Dutch Experiments on Animals Act (WOD) license AVD11500202115359. The procedures followed the recommendations outlined in the “Guide for the Care and Use of Laboratory Animals.” Throughout the study, the animals had unrestricted access to water and standard chow and were housed in a consistent environment, including a 12 h light/dark cycle. The animals were randomly assigned to different treatment groups and cages, and blinding of operators was implemented whenever feasible.

#### Intramyocardial administration

Female BALB/c mice (Charles River Laboratories, 18–28 g, 8–13 weeks old) were administered a single intramyocardial injection of 10 μL containing 4 μg of LNP-encapsulated firefly luciferase modRNA (biodistribution and cytokine measurement), 4 μg of LNP-encapsulated EGFP modRNA (histology), or PBS as a control, as in previous work.^11^ The animals used for biodistribution and cytokine measurement were sacrificed after 24 h (*n* = 4 animals per experimental group and *n* = 3 animals for the PBS control). The animals used for histology were sacrificed after 5 days (*n* = 3 animals per experimental group and *n* = 3 animals for the PBS control). The treatment groups received four different mRNA-LNP formulations: SM-102 LNPs, ALC-0315 LNPs, D-Lin-MC3-DMA LNPs, or C12-200 LNPs. Mice were anesthetized with an intraperitoneal injection of fentanyl (0.07 mg/kg), midazolam (6.67 mg/kg), and dexdomitor (0.67 mg/kg), followed by intubation and mechanical ventilation set to 180 breaths per minute with a 1:1 oxygen-to-air ratio. Body temperature was maintained at 37°C using a heating pad during surgery. A left lateral thoracotomy was performed to access the heart, and LNP formulations were injected into the left ventricular wall at a rate of 10 μL/min using a 30G needle attached to a catheter connected to a remote syringe pump (Pump 11 Elite Nanomite) loaded with a 25 μL Hamilton syringe (model 1702 RN SYR). To prevent leakage, the needle was left in place for 30 s post-injection before being withdrawn. Surgical incisions were then closed, and the mice received a subcutaneous injection of an antagonist mixture containing atipamezole hydrochloride (3.3 mg/kg), flumazenil (0.5 mg/kg), and buprenorphine (0.2 mg/kg) for pain relief. After recovery from anesthesia, the mice were extubated and returned to their regular clean housing cages.

#### Blood collection and serum processing

For the animals sacrificed 24 h after firefly luciferase mRNA LNP administration, the mice were placed under terminal anesthesia, and blood was collected from the orbital venous sinus using serum separation tubes (Sarstedt AG & Co. KG). The serum was separated by centrifugation (10,000 × *g*, 5 min, 4°C) and stored at −80°C until further analysis.

#### *Ex vivo* analysis of organ luciferase activity

For the animals sacrificed 24 h after LNP administration, luciferase activity in different organs was assessed. Twenty-four hours after LNP administration, animals treated with LNP-encapsulated luciferase modRNA were intraperitoneally injected with 2.5 mg of D-luciferin (Promega) in a total volume of 100 μL. After a 10 min period, the animals were euthanized by cervical dislocation, and their organs were collected, rinsed in DPBS, and analyzed for luciferase signal using the IVIS RT PhotonImager (Biospace Lab). Following imaging, the organs were snap-frozen in liquid nitrogen and stored at −80°C for subsequent analysis.

#### Tissue lysates preparation for luminescence analysis

For the animals sacrificed 24 h after LNP administration, tissue lysates were prepared from the liver, spleen, lungs, kidneys, and heart. After thawing and weighing 100 mg of each organ, the spleens and hearts were finely chopped, and all organs were then transferred into Bead Mill tubes (VWR International) containing reinforced 4–5 mm beads (Qiagen). Metallic tissue-lysing beads were used for spleen and heart samples, while ceramic beads were used for liver, lung, and kidney samples. To each milligram of tissue, 5 μL of 1× Cell Culture Lysis Reagent (Promega) supplemented with a protease/phosphatase inhibitor cocktail (Cell Signaling Technology) was added. After incubation for 30 min at 4°C, the tissues were homogenized using a Mini bead-beater (Bertin Technologies) at 5,000 rpm for two cycles of 30 s each, with a 20 s interval in between. Subsequently, the samples were centrifuged at 10,000 × *g* for 10 min at 4°C, and the supernatant was transferred to fresh tubes. To assess luciferase activity, 10 μL of the supernatant were pipetted into a white 96-well plate (Greiner), followed by the addition of 50 μL of luciferase assay reagent (Promega) via injector. The reaction mixture was incubated for 2 s, and luminescence was measured and integrated over a 10 s period using the Spectramax ID3 (Molecular Devices).

#### Histology

For histological analysis, mice were sacrificed 5 days after LNP administration. The mice were euthanized, and their organs were perfused with 10 mL of PBS via cannulation of the vena cava. Hearts were collected and incubated for 72 h in 4% paraformaldehyde. Fixed hearts were subsequently embedded in paraffin and transversally cut into 4 μm slices sequentially from the apex to the base, with the entire organ represented on microscope glass slides for histological analysis. From each heart, representative slices were selected for immunohistochemistry or H&E staining, following standard deparaffinization and rehydration steps. H&E staining was performed using the Leica HistoCore spectra ST. For immunohistochemistry, Ly6G antibody (1:100 O/*N* 4°C, BD Pharmingen, catalog number 551459), MAC-3 antibody (1:200 O/*N* 4°C, eBioscience, catalog number 14-5989-85), and CD3 antibody (ready-to-use O/*N* 4°C, Roche, catalog number 790–4341) were used. For each animal, to identify the highest level of cell infiltration corresponding to the injection site, at least three H&E-stained histological sections from different heart levels were analyzed. Based on this, immunostaining for Ly6G (neutrophils), MAC-3 (macrophages), and CD3 (T cells) were performed on alternating sections where maximal cell infiltration was identified. The section showing the maximum infiltration area per staining and per animal was selected for quantification and plotting. H&E-stained and immunohistochemically labeled heart sections were imaged using a Hamamatsu XR scanner (Hamamatsu, Japan; *n* ≥ 3 per staining/animal). Image analysis and quantification were performed in ImageJ using either a custom-made script (for H&E and Ly6G) or manual thresholding (for MAC-3 and CD3).

#### Inflammatory cytokine quantification and liver toxicity assessment

ProcartaPlex Mouse Th1/Th2 Cytokine Panel, 11-plex (Thermo Fisher Scientific) kits were utilized to measure the concentrations of various protein targets in the serum of all treated animals, following the manufacturer’s instructions. The protein targets measured included GM-CSF, IFN-γ, IL-1β, IL-2, IL-4, IL-5, IL-6, IL-12p70, IL-13, IL-18, and TNF-α. Serum samples were diluted 1:1 and analyzed in duplicate using a Luminex xMAP Bio-Plex 200 instrument (Bio-Rad). Additionally, the Mouse ALT ELISA Kit (ALT 1) (Abcam) and the Mouse AST ELISA Kit (AST) (Abcam) were employed to assess liver toxicity, following the manufacturers’ protocols. Serum samples were pre-diluted in PBS at ratios of 1:730 for the ALT ELISA kit and 1:640 for the AST ELISA kit.

### Statistical analysis

Statistical analysis was performed using GraphPad Prism version 10 (GraphPad Software). Differences between groups were tested using a two-tailed one-way ANOVA.

## Data and code availability

The data underlying this article are available in the article and in its online supplemental information.

## Acknowledgments

This work was supported by the Dutch Heart Foundation Dr. E. Dekker Senior Scientist grant, #2019T049, and the EXPERT project from the European Union's Horizon 2020 research and innovation program under grant agreement no. 825828. J.P.G.S. is supported by H2020-EVICARE (#725229) of the 10.13039/501100000781European Research Council (ERC), by ZonMw Psider-Heart (10250022110004), NWO-TTP HARVEY (2021/10.13039/501100024872TTW/01038252), and NWO_Era4Health-Cardinnov-080 RESCUE. We would like to thank Domenico Castigliego and Hans Möring for their technical assistance with heart section scanning and needle adaptation to the syringe pump device for intramyocardial administration, respectively. The graphical abstract was created using BioRender.com.

## Author contributions

Conceptualization, P.V.; formal analysis, M.C.I.L. and P.E.M.d.C.; funding acquisition, R.M.S., J.P.G.S., and P.V.; investigation, M.C.I.L., P.E.M.d.C., P.H.v.d.K., M.A.D.B, Q.Y., W.S.d.V.; methodology, M.C.I.L., P.E.M.d.C., Q.Y., Z.L.; supervision, S.C.A.d.J., R.M.S., J.P.G.S., and P.V.; writing – original draft, M.C.I.L, P.E.M.d.C., and P.V.; writing – review & editing: J.P.G.S. and P.V.

## Declaration of interests

The authors declare no competing interests.

## Supplemental information

Document S1. Figures S1–S3

Document S2. Article plus supplemental information
