# Supplementary material for: Efficiency and immunogenicity of lipid nanoparticle-mediated cardiac mRNA delivery are lipid composition-dependent
Source: Mol Ther Nucleic Acids. 2026 Apr 22;37(2):102939. doi: 10.1016/j.omtn.2026.102939 (PMC13191635; doi:10.1016/j.omtn.2026.102939)
Supplement: Document S1. Figures S1–S3 [file mmc1.pdf]

OMTN, Volume *37*

Supplemental information

Efficiency and immunogenicity of lipid nanoparticle-mediated cardiac mRNA delivery are lipid composition-dependent

Maria C.I. Labonia, Pol Escudé Martinez de Castilla, Petra H. van der Kraak, Maike A.D. Brans, Qiangbing Yang, Zhiyong Lei, Saskia C.A. de Jager, Willemijn S. de Voogt, Raymond M. Schiffelers, Joost P.G. Sluijter, and Pieter Vader

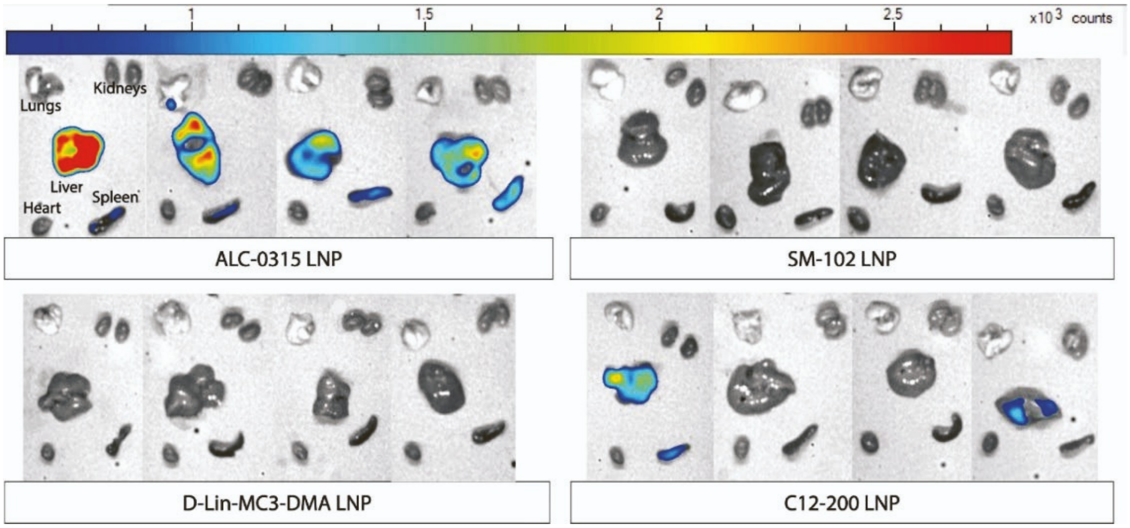

Figure S1 | Delivery efficiency of luciferase encoding modRNA measured by luminescence imaging of organs at 24 h. Organ luminescence imaged at higher counts per minute in order to show unsaturated signal in the ALC-0315 LNP treated mice organs. Four panels for each formulation represent four different mice.

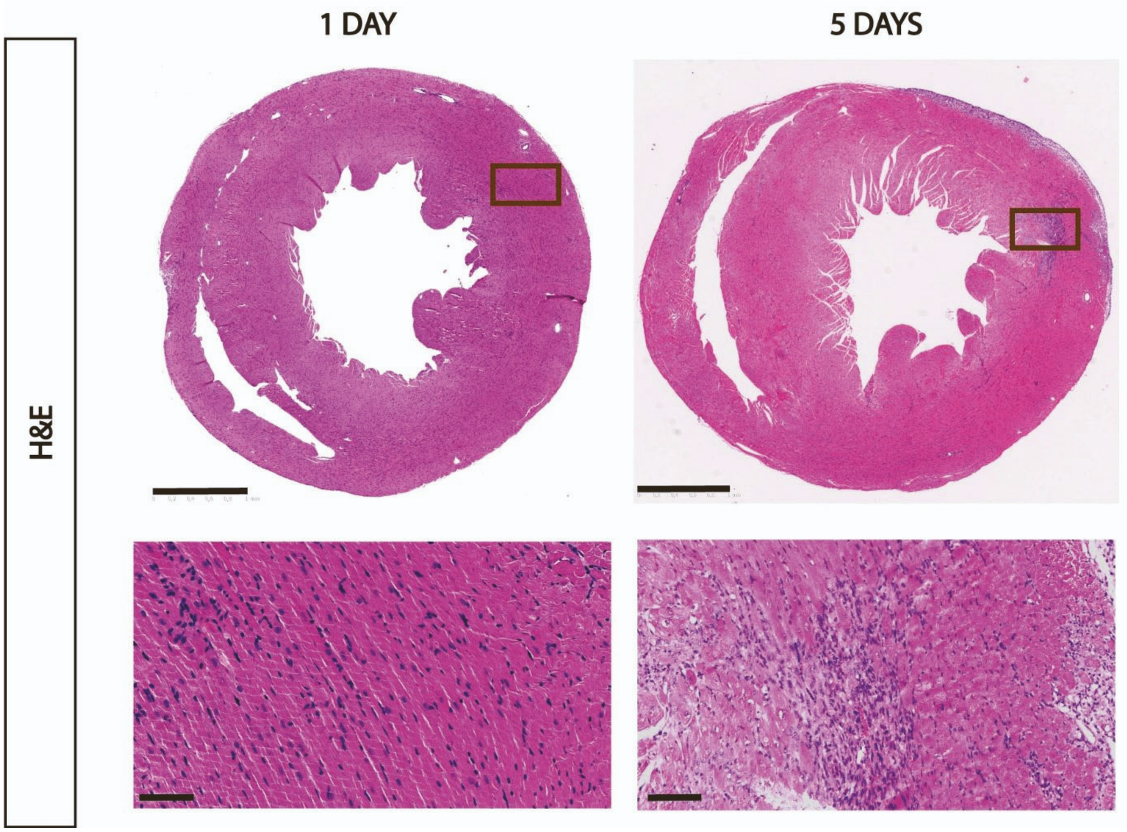

Figure S2 | SM-102 LNPs do not induce detectable local cell infiltration 1 day post-administration. H&E staining of transversal heart sections at 1 and 5 days following intramyocardial administration of SM-102 LNPs containing 4µg of LNP-encapsulated modRNA. The histological images highlight marked differences in the local immune response to mRNA-LNP administration over time. Representative regions of the treated myocardium are indicated by squares and shown at higher magnification below. Scale bars in the upper row represent 1 mm; scale bars in the magnified images represent 100 µm.

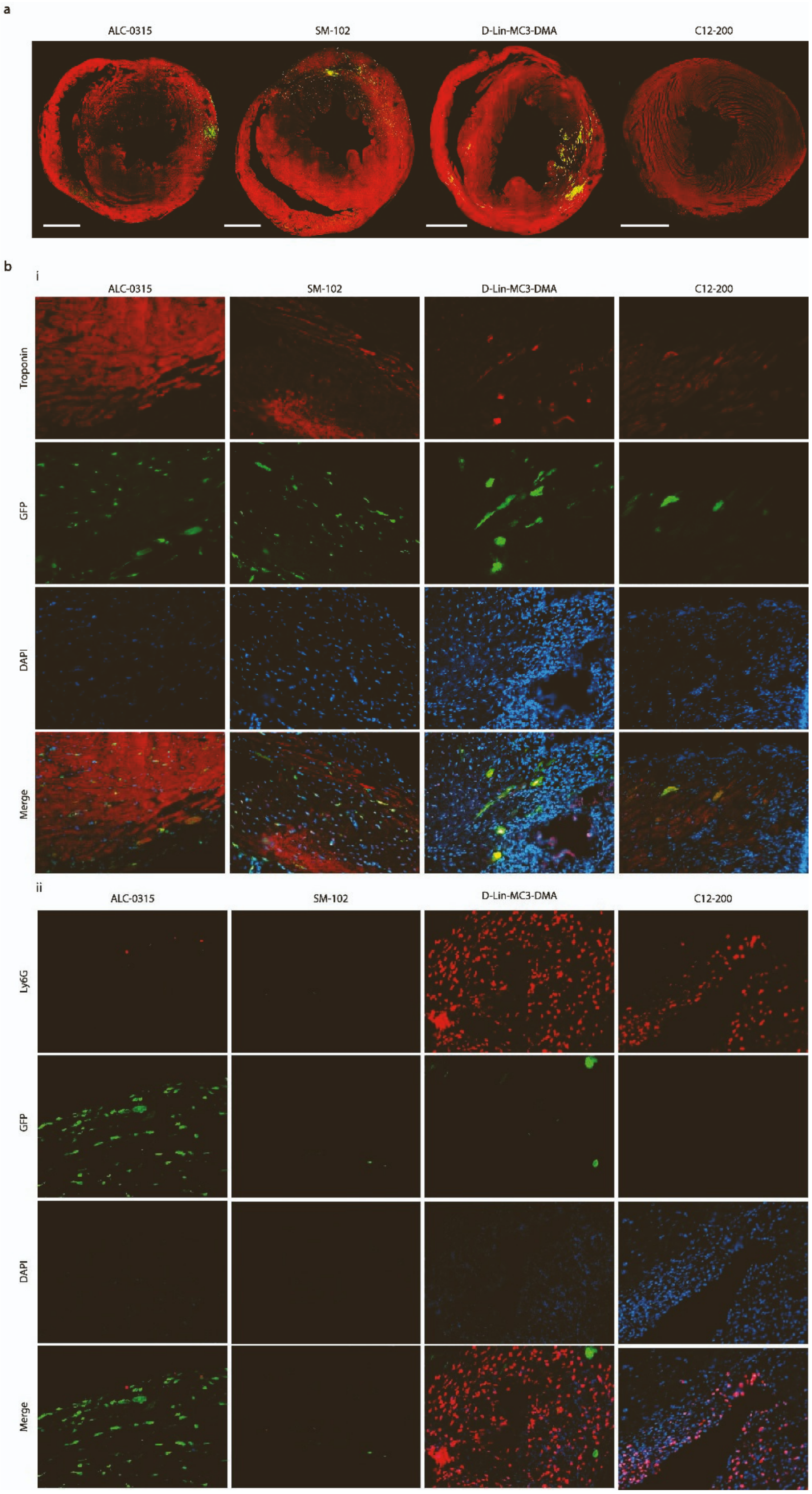

Figure S3 | ModRNA-LNPs transfect both cardiomyocytes and interstitial cells highly localized at the injection site. Immunofluorescence stainings of representative heart sections collected 5 days after local injection of 4 µg modRNA encapsulated in LNPs. (a) Complete transversal heart sections stained for troponin (in red) and GFP (in green). Scale bars represent 1mm. (b) 40× magnification of the localized treated areas showing GFP-expressing cell types (in green), with cardiomyocytes identified by troponin staining (in red) (i), neutrophils identified by Ly6G staining (in red) (ii) and cell nuclei identified by DAPI (in blue).
